# CD154:CD11b blockade enhances CD8^+^ T cell differentiation during infection but not transplantation

**DOI:** 10.1172/jci.insight.184843

**Published:** 2025-06-09

**Authors:** Katie L. Alexander, Kelsey B. Bennion, Danya Liu, Mandy L. Ford

**Affiliations:** 1 Emory Transplant Center, Department of Surgery, and; 2 Emory Department of Surgery, Emory University School of Medicine, Atlanta, Georgia, USA.

**Keywords:** Immunology, Transplantation, Costimulation, Organ transplantation, T cells

## Abstract

CD154 is a promising target for immunosuppression in transplantation, autoimmunity, and inflammatory diseases. We previously identified CD11b as a novel alternative receptor for CD154 during alloimmunity. However, the impact of specific CD154:CD11b blockade on immune responses to infection has not been well characterized. Here, we have shown that in contrast with its immunosuppressive effect on graft-specific CD8^+^ T cells, CD154:CD11b blockade unexpectedly improved both the quantity and quality of murine herpesvirus-68–specific CD8^+^ T cells as measured by an increase in tetramer-positive KLRG1^lo^CD127^hi^ memory precursor effector cells. The differential effect of CD154:CD11b blockade on graft- versus virus-specific CD8^+^ T cells was underpinned by differences in phosphorylated S6 downstream of mTOR complex 1; however, differential expression of key transcription factors Eomes and TCF-1 was dictated by the type of antigen stimulus. These data demonstrate that priming conditions play an important role in determining the outcome of immunotherapy and suggest that specific inhibition of CD154:CD11b interactions could be effective for suppressing alloimmune responses while maintaining protective immunity to minimize infectious complications following transplantation.

## Abstract 2

CD11b is a novel alternate receptor for CD154; however, the role of CD154:CD11b interactions in anti-viral immunity is unknown. This work shows that in contrast to its immunosuppressive effect on graft-specific CD8^+^ T cells, CD154:CD11b blockade unexpectedly augments anti-viral CD8^+^ T cell responses.

## Introduction

The CD154 costimulation pathway plays an important role in the activation of the adaptive immune response. Engagement of CD154 (CD40L) with the CD40 receptor results in the provision of T cell help for the induction of a germinal center response and B cell class switching (1). CD154:CD40 interactions also play a direct role in CD4^+^ T cell–mediated DC licensing to promote antigen presentation and priming of CD8^+^ T cells (2). Over multiple decades, the CD154 pathway has remained a promising therapeutic target to suppress unwanted immune responses, for example, promoting long-term graft survival for solid organ transplantation in both mouse and nonhuman primate models (3, 4). Under some conditions, CD154 pathway blockade has been shown to induce durable tolerance to allografts even after cessation of immunosuppression (5). In addition, blockade of CD154:CD40 interactions has also been shown to ameliorate idiopathic cytopenic purpura and more recently amyotrophic lateral sclerosis (6, 7). However, initial clinical trials with Fc-intact anti-CD154 mAbs revealed an association with thromboembolic complications, resulting in a search for alternatives (8–10). Attempts were made to target the receptor for CD154, CD40, with anti-CD40 mAbs that have shown promise in treating autoimmune diseases, such as systemic lupus erythematous and inflammatory bowel disease (11, 12). Unfortunately, in preclinical studies of renal transplantation in the nonhuman primate model, anti-CD40 mAbs were found to be inadequate at preventing graft rejection as a monotherapy, with significantly lower graft survival compared with anti-CD154 mAbs (13).

A potential explanation for the mechanistic basis underlying the observed difference in the efficacy of CD154 versus CD40 blockers is the possibility that CD154 could be interacting with a receptor other than CD40 to mediate alloimmunity. In a 2018 study, CD154 binding to the integrin CD11b (complexed with CD18: αMβ2, Mac-1) was implicated in the pathogenesis of atherosclerosis (14). Blockade of the CD154:CD11b interaction was achieved using a specific peptide mimetic of the CD154 binding domain on CD11b (cM7) that prevented this interaction without interfering with the ability of CD154 to bind to CD40 or with the ability of CD11b to bind to its other ligands (15, 16). Moreover, we previously demonstrated that CD154:CD11b interactions contribute to alloimmunity, in that blockade of both CD40 and CD11b interactions fully recapitulated the superior graft survival observed with CD154 blockade (17). We also established that the use of the CD154:CD11b-blocking peptide inhibitor was sufficient to block this interaction and improve survival in the context of CD40 blockade. These data established the blockade of the CD154:CD11b interactions as a potential adjunctive immunosuppressive therapy.

Given its potential for use as an immunomodulatory strategy to suppress auto- and alloimmunity, here we interrogated the impact of CD154:CD11b blockade on protective immunity to a viral infection. Current standard-of-care immunosuppression for use in transplantation renders patients more susceptible to infection, including a significant increase in reactivation of common latent viral infections that are typically controlled in immunocompetent individuals, such as cytomegalovirus and Epstein-Barr virus (EBV) (18). EBV is the causative agent of infectious mononucleosis and infects approximately 90% of humans by adulthood (19). Murine herpesvirus-68 (MHV68) is a well-established small animal model of EBV infection that allows for easy investigation of preclinical therapeutics (20).

Thus, in this study, we sought to address the impact of CD154:CD11b blockade alone on alloimmunity and protective immunity using murine models of skin transplantation and EBV infection. We found that CD154:CD11b blockade alone decreased CD8^+^ T cell responses elicited via transplantation. In contrast, not only did CD154:CD11b blockade not impair the anti-infection response, but it also unexpectedly increased both the quantity and quality of the CD8^+^ T cell response. These findings illuminate a dichotomy in CD8^+^ T cell differentiation programs elicited in response to a tissue- versus infection-derived antigen.

## Results

### Selective CD154:CD11b blockade alone inhibits alloreactive CD8^+^ T cell responses in the context of transplantation.

**Figure 1:**
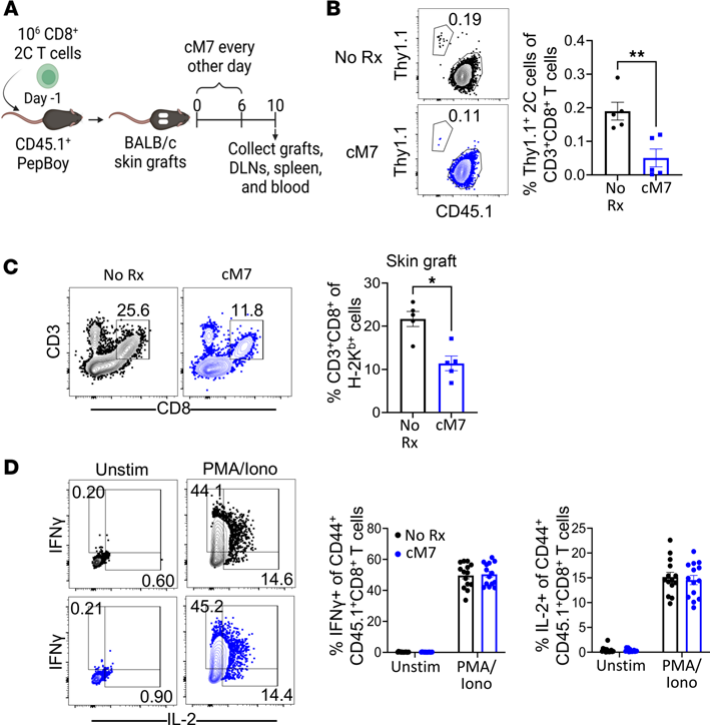
CD154:CD11b blockade alone attenuates the magnitude of the CD8^+^ T cell response to an allograft. (**A**) CD45.1^+^ congenic mice received 1 × 10^6^ BALB/c-specific TCR-transgenic 2C splenocytes and BALB/c skin grafts, as well as either no further treatment or cM7 on days 0, 2, 4, and 6. Skin grafts, draining lymph nodes (DLNs), spleen, and blood were collected 10 days posttransplantation. (**B**) Representative flow cytometry plots and summary data of frequencies of Thy1.1^+^ 2C cells of CD3^+^CD8^+^ T cells in the spleen. (**C**) Representative flow cytometry plots and summary data of frequencies of infiltrating CD3^+^CD8^+^ T cells of H-2Kb^+^ cells in skin allografts. (**D**) Representative flow cytometry plots and summary data of frequencies of IL-2– and IFN-γ–producing cells of CD44^+^CD8^+^ T cells following restimulation with PMA and ionomycin. Data are representative of 3 individual experiments with 5–14 mice/group. *P < 0.05, **P < 0.01 by Mann-Whitney test or 2-way ANOVA with Šidák’s test where appropriate.

Our previously published results showed that the addition of the CD154:CD11b blocker to anti-CD40 during allogeneic transplantation resulted in a further decrease in the number of graft-infiltrating cells and increased allograft survival as compared with anti-CD40 alone (17). However, the effect of CD154:CD11b blockade alone on the alloimmune response during transplantation was not studied. To investigate this, CD45.1^+^ congenic mice received 1 × 10^6^ BALB/c-specific TCR-transgenic 2C T cells and BALB/c skin grafts, as well as either no further treatment or the CD154:CD11b blocker cM7 on days 0, 2, 4, and 6 (Figure 1A). Skin grafts, DLNs, spleen, and blood were collected on day 10. A significant decrease in the frequency and number of graft-specific Thy1.1^+^CD8^+^ 2C T cells in the spleen was observed following cM7 treatment as compared with untreated controls (Figure 1B).

To determine if CD154:CD11b blockade alone exerted an effect on infiltration of lymphocytes into the allograft, as previously observed in combination with anti-CD40 treatment (17), frequencies of recipient-derived H-2Kb^+^CD3^+^CD8^+^ graft-infiltrating cells were determined for untreated versus cM7-treated skin graft recipients. Results indicated a significant decrease in the frequency of CD3^+^CD8^+^ graft-infiltrating T cells (among total H-2Kb^+^ recipient-derived cells) in the cM7-treated animals compared with untreated controls (Figure 1C).

Finally, we assessed the impact of selective CD154:CD11b blockade on the functionality of graft-specific CD45.1^+^CD8^+^ T cells in the context of transplantation (Figure 1D). Briefly, splenocytes were obtained from untreated versus cM7-treated skin graft recipients on day 10 posttransplantation and restimulated ex vivo. No significant differences in the frequency of both IFN-γ– and IL-2–producing CD44^+^CD45.1^+^CD8^+^ T cells were found between the untreated versus cM7-treated animals (Figure 1D). The impact of CD154:CD11b blockade on CD4^+^ T cells was investigated. Interestingly, a modest decrease in the frequency of CD44^+^CD4^+^ T cells 10 days posttransplantation was observed in the cM7-treated mice compared with untreated controls (Supplemental Figure 1A; supplemental material available online with this article; https://doi.org/10.1172/jci.insight.184843DS1). However, no significant impact of CD154:CD11b blockade on the production of IL-2 and IFN-γ by CD4^+^ T cells was observed (Supplemental Figure 1B).

CD154 pathway inhibition is known to also be beneficial in its ability to inhibit the generation of donor-specific antibody (DSA) and decrease antibody-mediated rejection (21). This is primarily a result of blocking CD154:CD40 interactions necessary for B cell activation and differentiation (22, 23). To determine the effect of blocking CD154:CD11b interactions on B cell responses, serum was collected 14 days posttransplantation for assessment of relative levels of DSA using flow cytometry–based crossmatching against BALB/c cells. CD154:CD11b-specific blockade had no significant impact on the generation of DSA according to both MFI and fold-change normalized to naive control serum (Supplemental Figure 2). These results support the conclusion that while alloreactive B cell responses are dependent on CD154:CD40 interactions, they are independent of CD154:CD11b interactions. The lack of observed differences on DSA and CD4^+^ T cells led us to focus on the impact of CD154:CD11b blockade on CD8^+^ T cells.

### CD154:CD11b blockade increases the quantity and quality of the virus-specific CD8^+^ T cell response.

**Figure 2:**
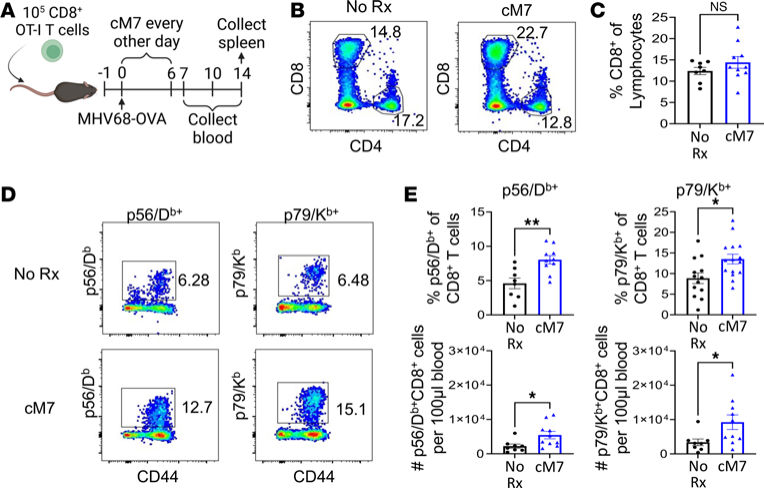
CD154:CD11b blockade increases the frequency and number of virus-specific CD8^+^ T cells. (**A**) WT C57BL/6 mice were infected with MHV68 and were untreated or treated with cM7 on days 0, 2, 4, and 6. Blood was collected on days 7, 10, and 14 with spleens collected on day 14. (**B** and **C**) Representative flow cytometry plots and summary data of frequencies of CD8^+^ T cells on day 10 in the blood. (**D** and **E**) Representative flow cytometry plots and summary data of frequencies and numbers of antigen-specific CD8^+^ T cells using MHC class I tetramers for 2 lytic cycle viral epitopes, p56 and p79, on day 10 in the blood. Data are representative of 3 individual experiments with 4–10 mice/group. *P < 0.05, **P < 0.01 by Mann-Whitney test.

The impact on protective immunity is a key consideration in the development of new immunosuppression and should be used to identify promising therapies that can prevent rejection with minimal impact on the protective immune response. To assess the impact of CD154:CD11b blockade on the CD8^+^ T cell response to infection, wild-type C57BL/6 mice were infected with 1 × 10^5^ PFU MHV68-OVA and left untreated or treated with the CD154:CD11b blocker cM7. Blood was collected on days 7, 10, and 14, and spleens were collected on day 14 for analysis (Figure 2A). No significant differences were observed in the frequency of either bulk or endogenous antigen-specific CD8^+^ T cells in the blood on day 7 in cM7-treated animals compared with untreated controls (not shown). By day 10, despite no significant difference in the frequency of bulk CD8^+^ T cells (Figure 2, B and C), cM7-treated mice exhibited a significant increase in both the frequency and absolute number of virus-specific CD8^+^ T cells identified using MHC class I tetramers of 2 lytic cycle viral epitopes, p56 and p79, as compared with untreated mice (Figure 2, D and E). TCR-transgenic antigen-specific OT-I T cells were also enumerated. Interestingly, the increase in frequency of antigen-specific CD8^+^ T cells observed on day 10 for the endogenous response during CD154:CD11b blockade was observed on day 7 for the OT-Is (Supplemental Figure 3). These findings are in sharp contrast with the observed decreases in graft-specific CD8^+^ T cells on day 10 posttransplantation (Figure 1B).

The impact of CD154:CD11b blockade was also investigated in CD4^+^ T cells. There was no observed difference in the frequency of CD44^+^CD4^+^ T cells (Supplemental Figure 1C) and no difference in the induction of IL-2 or IFN-γ production following in vitro restimulation (Supplemental Figure 1D). These data further indicate that the effect of CD154:CD11b blockade on T cells primarily affects the CD8^+^ T cell response.

**Figure 3:**
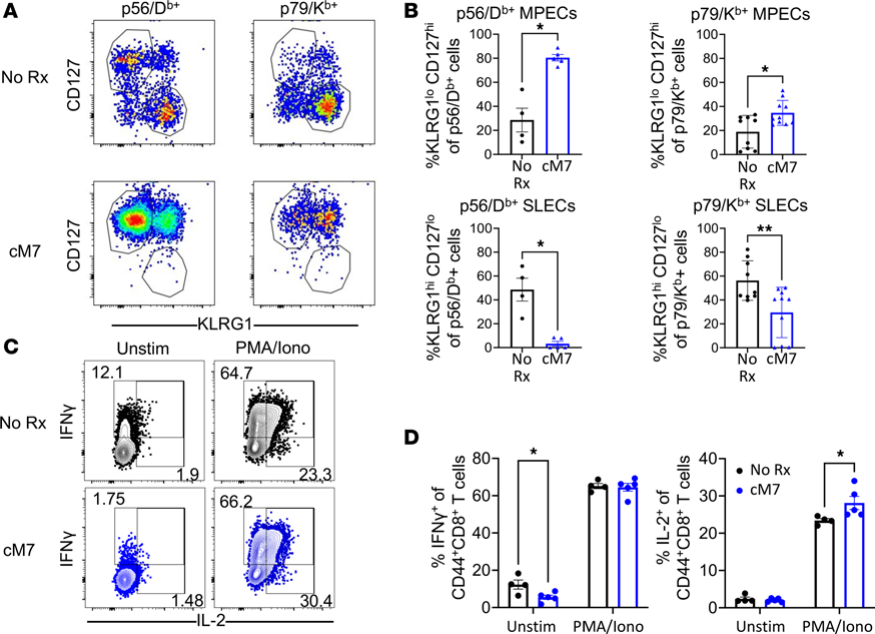
CD154:CD11b blockade improves the quality of the virus-specific CD8^+^ T cell response. WT C57BL/6 mice were infected with MHV68 and were untreated or treated with cM7 on days 0, 2, 4, and 6. Spleens were collected on day 14. (**A** and **B**) Representative flow cytometry plots and summary data of frequencies of KLRG1^lo^CD127^hi^ memory precursor effector cells (MPECs) and KLRG1^hi^CD127^lo^ short-lived effector cells (SLECs) on day 14 in the spleen. (**C** and **D**) Representative flow cytometry plots and summary data of frequencies of IL-2– and IFN-γ–producing cells of CD44^+^CD8^+^ T cells following restimulation with PMA and ionomycin. Data are representative of 3 individual experiments with 4–10 mice/group. *P < 0.05, **P < 0.01 by Mann-Whitney test or 2-way ANOVA with Šidák’s test where appropriate.

To understand the qualitative differences that might be associated with the unexpected increase in the magnitude of virus-specific CD8^+^ T cells in the animals in which CD154:CD11b interactions were blocked, we assessed the relative proportion of long-lived KLRG1^lo^CD127^hi^ memory precursor effector cells (MPECs) versus KLRG1^hi^CD127^lo^ short-lived effector cells (SLECs) in these animals (24). Results indicated that on day 14 in the spleen, a significant increase in the frequency of KLRG1^lo^CD127^hi^ MPECs among both p56/D^b^-specific and p79/K^b^-specific CD8^+^ T cell populations was observed in cM7-treated animals as compared with untreated controls. Likewise, a coordinate decrease in the frequency of KLRG1^hi^CD127^lo^ SLECs among both p56/D^b^-specific and p79/K^b^-specific CD8^+^ T cell populations was observed in the cM7-treated group compared with no treatment (Figure 3, A and B). To determine whether this increase in the frequency of MPECs was due to an increase in the number of MPECs or a decrease in the number of SLECs, absolute numbers of both populations were analyzed. Results demonstrated a significant increase in the number of MPECs within both the p56/D^b^- and p79/K^b^-specific CD8^+^ T cell populations in the cM7-treated animals as compared with untreated controls (Supplemental Figure 4A). However, the absolute number of MPECs for both tetramers was more pronounced and significantly increased in the cM7-treated animals as compared with untreated controls, suggesting a skewing toward MPECs beyond the impact of an increased parent population (Supplemental Figure 4B). These data suggest that the immunosuppressive peptide inhibitor cM7 functioned to preferentially increase the number of virus-specific long-lived memory precursors and thus increase both the quantity and quality of the CD8^+^ T cell response to infection.

To determine whether this increase in number and quality of virus-specific CD8^+^ T cells following CD154:CD11b blockade is also observed during broad CD154 pathway blockade, we treated animals with anti-CD154 (MR-1), which blocks the interaction of CD154 with both CD11b and CD40. In contrast with the effect of specific CD154:CD11b blockade, pan-CD154 blockade resulted in no significant difference in the frequency of CD8^+^ OT-I T cells on day 7 in the blood, and tetramer^+^ virus-specific cells of CD8^+^ T cells on day 10 in the blood, compared with untreated controls (Supplemental Figure 5, A and B). However, similar to the effect of specific CD154:CD11b blockade, pan-CD154 blockade resulted in a significant increase in the frequency of MPECs among tetramer^+^CD8^+^ T cells, and a coordinate decrease in the frequency of SLECs among tetramer^+^CD8^+^ T cells on day 14 in the spleen, as compared with untreated controls (Supplemental Figure 5C). Overall, these results indicate that CD154 pathway blockade in general skewed differentiation toward MPECs and away from SLECs, but only the more specific CD154:CD11b blockade also significantly increased the frequency of antigen-specific CD8^+^ T cells and thus the magnitude of the protective immune response.

To determine if CD154:CD11b blockade impaired the function of the CD8^+^ T cells in response to infection, we measured cytokine production 14 days postinfection following ex vivo restimulation. No significant differences in the frequency of IFN-γ–producing cells among CD44^+^CD8^+^ T cells was observed in the cM7-treated versus untreated groups. However, the frequency of IL-2–producing cells among CD44^+^CD8^+^ T cells was significantly increased in the cM7-treated group following restimulation compared with untreated controls (Figure 3, C and D). These results are consistent with the increased MPEC phenotype and suggest that CD154:CD11b blockade resulted in the generation of more multipotent, higher quality cytokine-producing, virus-specific CD8^+^ effectors following infection as compared with untreated controls.

### Control of MHV68 viral load is maintained during CD154:CD11b blockade.

**Figure 4:**
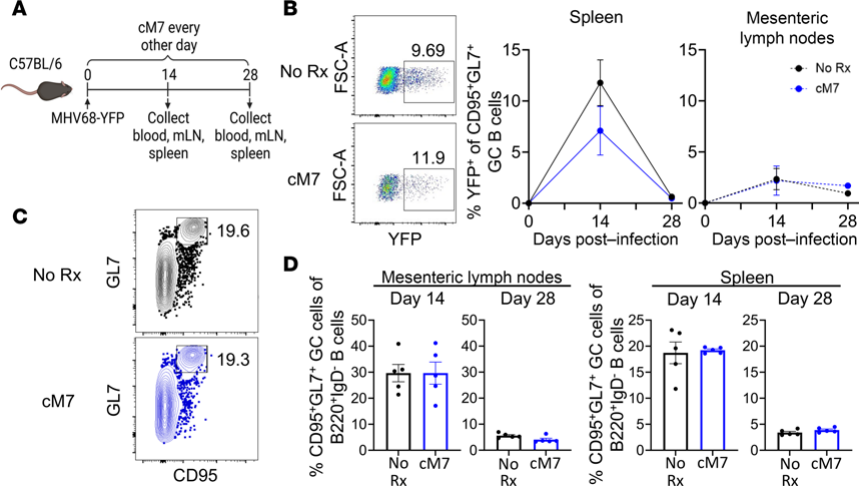
Control of viral load is maintained during CD154:CD11b blockade. (**A**) WT C57BL/6 mice were infected with MHV68 containing a fusion protein composed of the enhanced YFP-coding sequence (MHV68-YFP) and were untreated or treated with cM7 on days 0, 2, 4, and 6. Blood, mesenteric lymph nodes (mLNs), and spleen were collected on day 14 or 28. (**B**) Representative flow cytometry and summary data of frequencies of virally infected YFP^+^ cells of CD95^+^GL7^+^ GC B cells. (**C** and **D**) Representative flow cytometry and summary data of frequencies of CD95^+^GL7^+^ GC B cells of bulk, class-switched B220^+^IgD^–^ B cells. Data are from 1 representative replicate of 2 individual experiments with 5 mice/group. Mann-Whitney test.

The unexpected observation of an increased CD8^+^ T cell response following treatment with cM7 suggests that cM7 may not be deleterious for control of viral infection. Therefore, we interrogated the control of MHV68 viral load during CD154:CD11b blockade using the inhibiting peptide, cM7. To accomplish this, we utilized an MHV68 virus engineered to express yellow fluorescent protein (YFP) such that virally infected cells can be measured by flow cytometry (25). This virus has previously been shown to mark infected cells early during the establishment of latency (26), facilitate the identification of changes in infected cell populations when specific viral genes are knocked out (27), allow the tracking of populations of infected cells in knockout strains of mice (28), and facilitate the monitoring of infection in vitro (29, 30). Wild-type mice were infected with 1 × 10^5^ PFU MHV68-YFP and left untreated or treated with cM7 every other day. Blood, mesenteric lymph nodes (mLNs), and spleen were collected on day 14 during peak viral load and day 28 upon resolution of acute infection (Figure 4A). MHV68 primarily infects germinal center (GC) B cells and is capable of interfering with the normal proliferative programming of these cells; thus, YFP expression was assessed in B220^+^IgD^–^CD95^+^GL7^+^ GC B cells (31, 32). Both untreated and cM7-treated groups were able to maintain control of viral load, as evidenced by a significant drop in frequency of YFP^+^ GC B cells by day 28 in the spleen, the primary reservoir of infection (31), with maintenance of a small frequency of YFP^+^ GC B cells in the mLNs (Figure 4B). The frequency of CD95^+^GL7^+^ GC B cells among total B220^+^IgD^–^ B cells did not significantly differ between treated versus untreated groups in the spleen and mLNs at either the day 14 or 28 time point (Figure 4, C and D). Therefore, both groups maintained a similar ability to control aberrant proliferation in the GC that resulted from MHV68 infection. Bulk CD3^–^CD19^+^ B cells were also examined to ensure there was no aberrant proliferation among the bulk population. The cM7-treated groups exhibited a modest increase in the frequency of bulk CD3^–^CD19^+^ B cells among lymphocytes on day 14, which then dropped to significantly lower than untreated on day 28 in the mLNs and remained significantly lower at both time points for the spleen (Supplemental Figure 6). There was no significant difference in the frequency of YFP^+^ cells among bulk B cells at either time point in either mLNs or spleen (not shown). Overall, these data suggest that control of MHV68 viral load is maintained under CD154:CD11b blockade.

### CD154:CD11b blockade promotes a distinct transcriptomic profile in virus-specific CD8^+^ T cells.

**Figure 5:**
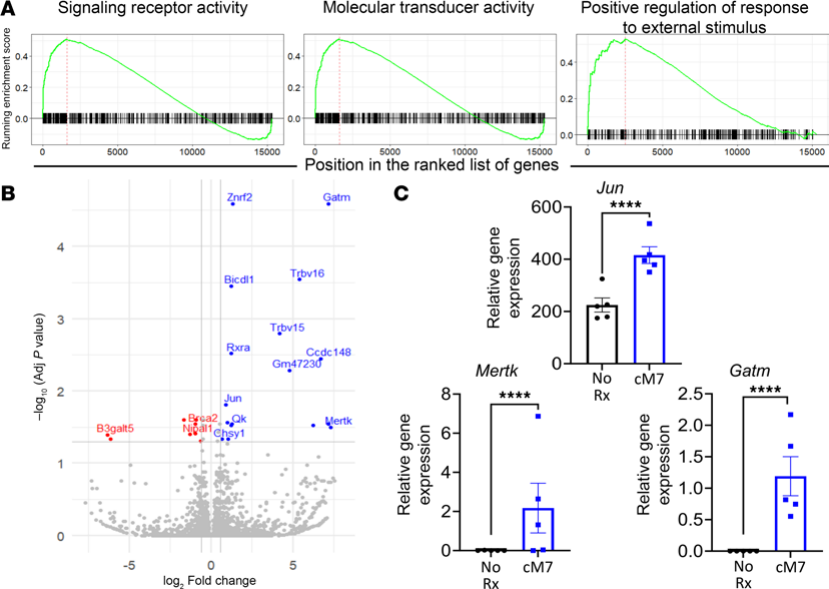
CD154:CD11b blockade promotes a distinct transcription profile in virus-specific CD8^+^ T cells. C57BL/6 mice were infected with MHV68 and left untreated or treated with cM7 on days 0, 2, 4, and 6. Bulk RNA-Seq was performed on FACS-sorted CD8^+^p79/K^b^ tetramer^+^ antigen-specific T cells from the spleen 10 days postinfection. (**A**) GSEA was conducted using clusterProfiler. GSEA plots are shown for the gene sets significantly enriched in the cM7-treated group. (**B**) Differential expression analysis was performed using DESeq2. Volcano plot is shown indicating the 25 significantly differentially expressed genes as upregulated (blue) or downregulated (red) in the cM7-treated group with *P* < 0.05 and fold-change > 1.5. (**C**) Relative gene expression shown as counts per million (CPM) values for genes of interest. Data are representative of 3 males and 2 females/group for a total of 5 mice/group. ****P < 0.0001 by Wald’s test with Benjamini-Hochberg correction.

To further explore the gene expression profile of the virus-specific CD8^+^ T cells in the context of CD154:CD11b blockade, WT mice were infected with MHV68 and either left untreated or treated with cM7 on days 0, 2, 4, and 6 as described in Methods. Bulk RNA-Seq was performed on day 10 FACS-sorted CD8^+^p79/K^b^ tetramer^+^ antigen-specific T cells. Gene set enrichment analysis (GSEA) identified 3 pathways that were significantly enriched in the cM7-treated group compared with the untreated group: signaling receptor activity (GO:0038023), molecular transducer activity (GO:0060089), and positive regulation of response to external stimulus (GO:0032103) (Figure 5A). All three are generally associated with an increase in cellular activation. Differential gene expression analysis was then conducted, and 25 genes were found to be significantly differentially expressed in the cM7-treated group versus the untreated group (Figure 5B). Several genes of interest were identified as significantly upregulated in the cM7-treated group versus untreated that are known to be associated with pathways significant in T cell biology: *Jun*, *Mertk*, and *Gatm* (Figure 5C). Taken together, this transcriptomic analysis suggests that blockade of CD154:CD11b interaction results in a distinct gene expression profile in virus-specific CD8^+^ T cells, potentially associated with higher quality cells.

Overall, these data indicate that despite CD154:CD11b blockade improving the quality and quantity of the CD8^+^ T cell response to viral infection, CD154:CD11b blockade attenuates the quantity of the graft-specific response.

### Differential expression of Eomes and TCF-1 transcription factors in antigen-specific CD8^+^ T cells elicited via a graft versus virus are not altered by CD154:CD11b blockade.

The data depicted above showed that CD154:CD11b blockade was able to augment virus-specific CD8^+^ T cell responses, with an underlying increase in the differentiation of MPECs. To determine if this effect was limited to the context of infection, MPEC and SLEC phenotypes were investigated in the allogeneic transplantation model. Of note, in the context of transplantation, virtually all the graft-specific Thy1.1^+^CD8^+^ 2C T cells were KLRG1^lo^CD127^hi^ MPECs (Supplemental Figure 7A); there were essentially no KLRG1^hi^CD127^lo^ SLECs among graft-specific Thy1.1^+^CD8^+^ 2C T cells in the spleen in both the cM7-treated and untreated groups (Supplemental Figure 7A). Thus, these findings reveal a fundamental difference in the relative proportion of MPECs versus SLECs within the antigen-specific CD8^+^ T cell response to a graft versus a microbe that is differentially impacted by CD154:CD11b blockade. To further investigate this phenomenon, we developed a direct comparison model where mice received an adoptive transfer of OT-I T cells and received either an OVA-expressing infection (MHV68-OVA) or skin graft (mOVA). The frequency of KLRG1^hi^CD127^lo^ SLECs in both the OT-I and bulk CD44^+^ populations 10 days after antigen stimulation verify that this model effectively recapitulates previously observed differences with a significant presence of SLECs following MHV68-OVA infection and virtually none following mOVA skin transplantation (Supplemental Figure 7B).

**Figure 6:**
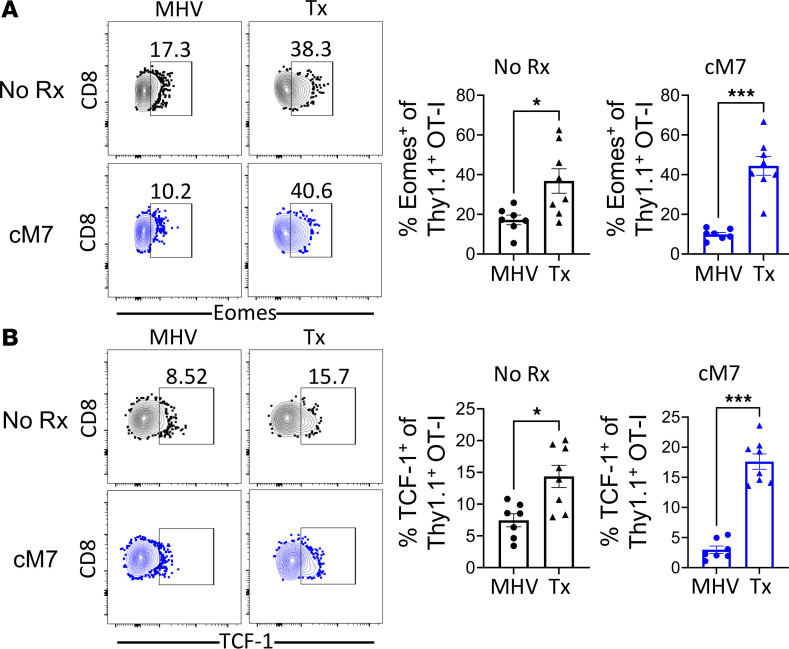
Antigen stimulus by infection or transplantation induces baseline differences in antigen-specific CD8^+^ T cell differentiation. WT C57BL/6 mice received TCR-transgenic OT-I T cells and were infected with MHV68-OVA or received OVA-expressing skin grafts. Spleens were collected on day 10 and processed for transcription factor staining. (**A**) Representative histograms and summary data of frequency of Eomes^+^ cells of CD8^+^ OT-I T cells. (**B**) Representative flow cytometry and summary data of frequency of TCF-1^+^ cells of CD8^+^ OT-I T cells. Data are representative of 3 individual experiments with 5–6 mice/group. *P < 0.05, ***P < 0.001 by Mann-Whitney test.

Thus, we next interrogated the transcription factor profile that underlies these distinct differentiation programs. Once again, WT mice received an adoptive transfer of OT-I T cells and received either an MHV68-OVA infection or mOVA skin graft. Spleens were collected 10 days after infection or transplantation. Results indicated that expression of Eomes and TCF-1, transcription factors known to be associated with MPEC differentiation, were significantly increased in the transplantation group compared with infection (Figure 6, A and B). There was no significant difference in induction of Eomes and TCF-1 following cM7 treatment, and the differences induced by infection versus transplantation exerted a dominant effect (Figure 6, A and B). Overall, these data suggest that the context of antigenic stimulation alters the differentiation program of CD8^+^ T cells during an immune response.

### Differential phosphorylation of S6 downstream of mTOR complex 1 in antigen-specific CD8^+^ T cells elicited via a graft versus virus is differentially altered by CD154:CD11b blockade.

**Figure 7:**
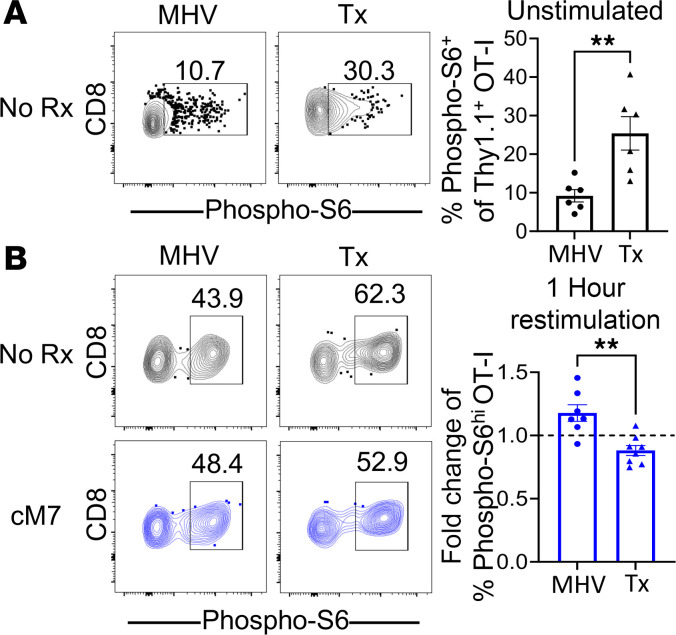
CD154:CD11b blockade differentially alters S6 phosphorylation in infection versus transplantation. (**A**) WT C57BL/6 mice received TCR-transgenic OT-I T cells and were infected with MHV68-OVA or received OVA-expressing skin grafts. Spleens were collected on day 10 and immediately fixed in 2% paraformaldehyde for phosphoflow (BD Biosciences). Representative flow cytometry and summary data of frequency of phosphorylated S6^+^CD8^+^ OT-I T cells are shown. Data are representative of 3 individual experiments with 5–6 mice/group. (**B**) WT C57BL/6 mice received TCR-transgenic OT-I T cells and were infected with MHV68-OVA or received OVA-expressing skin grafts. Spleens were collected on day 10, plated, and left unstimulated or restimulated for 1 hour with OVA peptide in vitro. Representative flow cytometry and summary data of the fold-change of cM7-treated over untreated of frequency of phosphorylated S6^hi^CD8^+^ OT-I T cells. Data are from 1 individual experiment with 7–8 mice/group. *P < 0.05, **P < 0.01 by Mann-Whitney test.

The RNA-Seq results shown in Figure 5 identified *Mertk*, as well as *Gatm*, as being differentially expressed following CD154:CD11b blockade during MHV68 infection. Both are known to activate mTOR complex 1 (mTORC1) signaling in CD8^+^ T cells (33–35). In addition, the induction of Eomes and TCF-1, found to be altered in transplantation versus infection in Figure 6, is known to be mediated by mTOR signaling, where a decrease in mTORC2 signaling favors the MPEC phenotype (36). S6 kinase is an immediate downstream product of mTORC1 that phosphorylates S6 and negatively regulates mTORC2 (37). To determine if differential antigen stimulation or cM7 treatment results in increased mTORC1 signaling, we investigated S6 phosphorylation in the OT-I comparison model. Corresponding with the observed differences in transcription factor induction in Figure 6, the frequency of phospho-S6^+^ OT-Is was significantly increased at a basal level during the graft-elicited response compared with the infection-elicited response 10 days after antigen encounter (Figure 7A). We also compared the impact of CD154:CD11b blockade with the addition of cM7 in the graft versus infection OT-I comparison. On day 10 after infection or transplantation, splenocytes were plated and restimulated for 1 hour before S6 phosphorylation assessment. Interestingly, CD154:CD11b blockade exerted a significantly differential impact on S6 phosphorylation in OT-I T cells elicited in the context of graft versus infection. Specifically, CD154:CD11b blockade resulted an increase in the frequency of phosphorylated S6^hi^ OT-I T cells compared with untreated mice during infection; in contrast, it resulted in a decrease of these cells during transplantation (Figure 7B). These results verify the potential downstream implication of the transcriptomic findings for *Mertk* and *Gatm* in the RNA-Seq dataset during infection. Overall, these data provide a potential mechanism underlying the observation that CD154:CD11b results in an increase in activation and survival of antigen-specific CD8^+^ T cells during infection but a decrease in these cells during transplantation.

## Discussion

The data presented here demonstrate that protective immunity is maintained during CD154:CD11b blockade, in contrast with its immunosuppressive effect on alloimmunity. CD154:CD11b blockade increases the frequency and number of antigen-specific CD8^+^ T cells during murine EBV infection, promotes the differentiation of MPECs, and promotes the production of the pro-survival cytokine IL-2. Virus-specific CD8^+^ T cells that develop in the presence of CD154:CD11b blockade also possess a transcriptional and expression profile associated with an increase in survival, including increased phosphorylation of S6 downstream of the mTORC1 pathway. This is in contrast with the immunosuppressive effect of CD154:CD11b blockade on graft-specific CD8^+^ T cells during transplantation, insofar as a decrease in the frequency and number of graft-specific CD8^+^ T cells is observed. These data reveal distinct roles for CD154:CD11b interactions during infection versus transplantation.

We posit that the observed difference in the effect of CD154:CD11b blockade is explained at least in part by baseline differences in the differentiation programs elicited via either a graft or a transplant. Specifically, transplantation elicited a population of CD8^+^ T cells that was approximately 90% MPECs, while the virus elicited a population of CD8^+^ T cells that were only approximately 20% MPECs. These altered differentiation programs were underpinned by differential phosphorylation of the S6 ribosomal protein downstream of mTORC1. Expression of TCF-1 was found to be consistent with the observed differences in MPEC differentiation during transplantation versus infection. However, this expression was not affected by CD154:CD11b blockade, suggesting that its effect is not dependent on differential expression of TCF-1. Instead, we speculate that CD154:CD11b blockade mediates its effect by attenuating the differentiation of antigen-specific CD8^+^ T cells. It may do this by reducing signaling via CD11b on antigen-presenting cells (APCs), thus altering the activation status of the CD8^+^ T cells. Indeed, we have previously shown in an in vitro culture system that cM7 inhibits the activation of CD11c^+^ DCs (38). During viral infection, this attenuation of APC activation may decrease the dominance of short-lived cells, promoting the MPEC phenotype and the persistence of virus-specific CD8^+^ T cells. In contrast, in the setting of transplantation, the antigen-specific CD8^+^ cell population already contains mostly MPECs, indicating little capacity for any increase in MPEC differentiation that could be induced by CD154:CD11b blockade. We posit that as a result, further attenuation of differentiation by CD154:CD11b blockade results in suboptimal activation and decreased accumulation of graft-specific effector CD8^+^ T cells.

This paradigm thus invokes that initial priming conditions play an important role in determining the effect of immune modulation and aligns with published literature that has identified other specific pathways that have differential effects during immune responses to transplantation versus infection. For example, rapamycin has previously been found to improve the response to *Listeria monocytogenes* while paradoxically preventing accumulation of graft-specific CD8^+^ T cells (39). Rapamycin treatment during lymphocytic choriomeningitis infection also significantly increased differentiation of the MPEC phenotype (40). In addition, coronin-1 blockade was shown to exert similar paradoxical effects where proliferation of graft-specific T cells was impaired without affecting the replicative potential in response to infectious stimuli, including salmonella and murine cytomegalovirus (41). The precise identity of the altered signal(s) that affect activation/differentiation in the context of these distinct stimuli remains unknown. However, because CD8^+^ T cell activation during viral infection involves the contribution of more signals from pattern recognition receptors (PRRs) as opposed to the sterile immune activation in the context of transplantation, it is tempting to speculate that these PRR/TLR signals may play a role. Identification of this mechanism will further our understanding of how targeted immunosuppression might be used to attenuate alloimmunity while maintaining protective immunity and minimizing the risk of infectious complications in transplant recipients. Further investigation is warranted in this regard.

Although our results demonstrated an increase in the quantity and quality of virus-specific CD8^+^ T cells in animals treated with CD154:CD11b blockade, this was not associated with a reduction in viral load. However, the observation of the lack of an increase in viral load in animals receiving CD154:CD11b blockade is still useful information for the field, insofar as an increase in viral load was observed in this mouse model of MHV68 infection in the setting of CD28 costimulation blockade with abatacept (42). Thus, the findings of preserved viral control in the setting of CD154:CD11b blockade may have important implications for the clinical development of CD154:CD11b blockers for use as an adjunctive therapy that reduces alloreactive T cells without further compromising protective immunity. We further speculate that the increase in CD8^+^ T cell response observed in the context of CD154:CD11b blockade may not be enough to result in reduction in viral load in an otherwise immunocompetent setting in which virus is largely controlled; however, it is possible that the increase in high-quality memory T cells may prove advantageous for better viral control over time or in the face of recurrent infection. Finally, the impact of pan-CD154 blockade on viral control in the MHV68 model has not been studied; future investigation to determine whether anti-CD154 would similarly confer preserved antiviral immunity would be beneficial in this regard.

Previous studies have examined the effect of pan-CD154 blockade (using an anti-CD154 mAb) on the immune responses to both transplantation and infection (3, 43). Similar to the results of CD154:CD11b blockade on the graft-specific immune response described here, anti-CD154 has been widely reported to have an immunosuppressive effect on graft-specific T cell responses (4). In contrast with the enhancing effect of specific CD154:CD11b blockade on microbe-specific CD8^+^ T cell responses shown here, blockade of CD154:CD40 interactions has been reported to have no effect on the frequency or number of *Listeria*-specific CD8^+^ T cells (44). These data are consistent with the lack of effect of an anti-CD154 mAb on the magnitude of CD8^+^ T cell response to virus shown here (Supplemental Figure 4, A and B). Based on these previously reported data along with our current results, we conclude that the increase in CD8^+^ T cell immunity may be specific to CD154:CD11b blockade and may not be observed when CD154:CD40 interactions are also blocked (potentially due to the concurrent loss of CD40 signaling). This conclusion has implications for the clinical development of selective CD154:CD11b inhibitors versus anti-CD154 mAbs, which block the interactions of CD154 with all its ligands.

## Methods

### Sex as a biological variable.

Our study examined both male and female mice and found no differences. Both sexes are represented in data from pooled experiments.

### Mice.

Male and female C57BL/6NCrl (B6) mice were obtained from the NIH/National Cancer Institute at Charles River Laboratories at 6–8 weeks old. Male B6.SJL-*Ptprc^a^Pepc^b^*/BoyCrCrl (CD45.1^+^ congenic PepBoys) and BALB/cAnNCrl (BALB/c) mice were obtained from the NIH/National Cancer Institute at Charles River Laboratories at 6–8 weeks old. TCR-transgenic B6.129S6-*Rag2^tm1Fwa^* Tg(TcraTcrb)1100Mjb (OT-I) mice were originally purchased from Taconic Farms, bred to the Thy1.1^+^ background, and maintained at Emory University. TCR-transgenic 2C mice that Sha et al. previously described were maintained at Emory University (45). All animals were housed in specific pathogen–free animal facilities at Emory University, with infected mice housed in Biosafety Level 2 animal facilities.

### Skin transplantation.

2C T cells were collected from spleens, and frequency was determined by staining with anti-CD8 (clone 53-6.7) and anti-Vβ13 (clone MR12-3) (both BD Biosciences) prior to adoptive transfer. Recipient mice received an adoptive transfer of 1 × 10^6^ 2C transgenic T cells 1 day before transplantation. Full-thickness ear and tail skins were transplanted bilaterally onto the dorsal thorax of recipient mice and secured with adhesive bandages as previously described (46).

### Viral infections.

For antigen-specific CD8^+^ T cell experiments, mice were infected i.p. with 1 × 10^5^ PFU of a transgenic strain of MHV68 containing a fusion of the transferrin transmembrane domain and OVA inserted at the v-cyclin locus (MHV68-OVA) (47). For viral load experiments, mice were infected i.p. with 1 × 10^5^ PFU of a transgenic strain of MHV68 containing a fusion protein composed of the enhanced YFP-coding sequence fused to the histone H2b open reading frame (MHV68-YFP) (25). Both strains were provided by Samuel Speck, Emory University.

### In vivo antibody and peptide treatment.

For MHV68-OVA infection and skin grafts, mice were treated with an i.p. injection of 100 μg of the CD154:CD11b peptide antagonist cM7 (C-EQLKKSKTL-C) (from GenScript) on days 0, 2, 4, and 6. In some experiments, mice were treated i.p. with 250 μg anti-CD154 (clone MR-1) (Bio X Cell) on days 0, 2, 4, and 6. For MHV68-YFP viral load experiments, mice were treated i.p. with 100 μg of cM7 every other day until day 26 with an experimental endpoint on day 28.

### Flow cytometry.

For skin graft experiments, blood, spleen, draining (brachial and axillary) lymph nodes (DLNs), and skin grafts were collected 10 days posttransplantation. Blood, spleen, and DLNs were processed as described above. Entire skin grafts were collected, cut into approximately 2 mm pieces, and digested using Collagenase P (Sigma-Aldrich) in HBSS with Ca^2+^ and Mg^2+^ (Corning) for 30 minutes at 37°C before homogenization through 70 μm, 40 μm, and 35 μm filters (VWR). LIVE/DEAD Fixable Aqua Dead Cell Stain Kit (Invitrogen) was used to differentiate live cells. All samples were stained with CD3-BUV737 (clone 17A2), CD4-BUV496 (clone GK1.5), and CD8-BUV805 (clone 53-6.7) from BD Biosciences, as well as CD45.1-BV605 (clone A20), Thy1.1-PerCP (clone OX-7), H2-K^b^–PE (clone AF6-88.5), KLRG1-BV421 (clone 2F1), and CD127-APC (clone A7R34) from BioLegend. Flow cytometric analysis was conducted using an LSRFortessa (BD Biosciences).

For MHV68-OVA infections, blood was collected on days 7, 10, and 14 postinfection and incubated with red blood cell High Yield Lyse Buffer (Invitrogen). Spleens were collected on day 14 and processed to a single-cell suspension through 70 and 40 μm cell strainers (Thermo Fisher Scientific). All samples were stained with CD4-Pacific Blue (clone GK1.5), CD8-BV785 (clone 53-6.7), Thy1.1-PerCP (clone OX-7), CD44–Alexa Fluor 700 (clone IM7), KLRG1-BV711 (clone 2F1), and CD127–PE-Cy7 (clone A7R34) (BioLegend), as well as p56/D^b^-APC and p79/K^b^-PE MHC tetramers from the NIH Tetramer Core. Flow cytometric analysis was conducted using an LSR II (BD Biosciences).

For MHV68-YFP infections, blood, spleen, and mLNs were collected 14 and 28 days postinfection. Blood and spleen were processed as described above, and mLNs were processed the same as spleens. Samples were stained with CD3–Alexa Fluor 700 (clone 17A2), CD19–PE-Cy7 (clone 6D5), CD95-APC (clone SA367H8), and CD138-PE (clone 281-2) from BioLegend. Flow cytometric analysis was conducted using an LSR II (BD Biosciences).

### Intracellular cytokine staining.

Following processing into a single-cell suspension, 2 × 10^6^ splenocytes were plated in a 96-well plate in complete R10 media and incubated for 4 hours with GolgiPlug (Brefeldin A) (BD Biosciences) and left unstimulated or stimulated with 20 ng/mL PMA and 1 μM ionomycin (both from Sigma-Aldrich). For MHV68-OVA experiments, cells were surface-stained with CD4–APC-Cy7 (clone RM4-5) from BD Biosciences and CD8-BV785 (clone 53-6.7), Thy1.1-PerCP (clone OX-7), BV605-Thy1.2 (clone 30-H12), and CD44–Alexa Fluor 700 (clone IM7) from BioLegend. For skin graft experiments, cells were surface-stained with LIVE/DEAD Fixable Aqua Dead Cell Stain Kit from Invitrogen; CD3-BUV737 (clone 17A2), CD4-BUV496 (clone GK1.5), and CD8-BUV805 (clone 53-6.7) from BD Biosciences; as well as CD45.1-BV605 (clone A20), Thy1.1-PerCP (clone OX-7), and CD44–Alexa Fluor 700 (clone IM7) from BioLegend. Cells were fixed and permeabilized using the Cytofix/Cytoperm Kit (BD Biosciences) according to the manufacturer’s instructions. Last, cells were stained intracellularly for IFN-γ–BV421 (BD Biosciences; clone XMG1.2) and IL-2–FITC (BioLegend; clone JES6-5H4). Flow cytometric analysis was conducted using an LSR II for infection experiments and an LSRFortessa for skin graft experiments.

### Phosphorylation-specific flow cytometry.

OT-I splenocytes were collected and processed to a single-cell suspension. A total of 2 × 10^6^ splenocytes were plated in a 96-well plate, left untreated or treated with 80 μg/mL cM7 peptide (GenScript), and stimulated with 1 nM OVA_257-264_ (SIINFEKL) (GenScript) for 1 hour. Following incubation, cells were stained with LIVE/DEAD Fixable Aqua Dead Cell Stain Kit, then stained with CD3-BUV737 (clone 17A2), CD4-BUV496 (clone GK1.5), and CD8-BUV805 (clone 53-6.7) from BD Biosciences and MERTK-BV421 (clone 2B10C42) from BioLegend. Cells were fixed in 4% paraformaldehyde (Beantown Chemical) and permeabilized using 90% methanol. Cells were rehydrated using 2% FBS in PBS and stained intracellularly for phosphorylated S6–Alexa Fluor 488 (Cell Signaling Technology; clone D57.2.2E). Flow cytometric analysis was conducted using an LSRFortessa.

### Transcription factor staining.

Following processing into a single-cell suspension, cells were stained with LIVE/DEAD Fixable Aqua Dead Cell Stain Kit to differentiate live cells. All samples were stained with CD3-BUV737 (clone 17A2), CD4-BUV496 (clone GK1.5), and CD8-BUV805 (clone 53-6.7) from BD Biosciences, as well as Thy1.1-PerCP (clone OX-7), CD44–APC-Cy7 (clone IM7), Tim-3–BV786 (clone RMT3-23), and PD-1–PE-Dazzle (clone 29F.1A12) from BioLegend. Samples were permeabilized using the Foxp3/Transcription Factor Staining kit (eBioscience) and stained with Eomes-BUV395 (clone X4-83) and TCF-1/7–Alexa Fluor 647 (clone S33-966) from BD Biosciences. Flow cytometric analysis was conducted using an LSRFortessa.

### Cell sorting and RNA-Seq.

Splenocytes were collected from wild-type mice left untreated or treated with cM7 10 days postinfection and processed to a single-cell suspension as described above. Samples were enriched for lymphocytes using a density gradient centrifugation with Ficoll-Paque PREMIUM 1.084 (Sigma-Aldrich) according to the manufacturer’s instructions. Cells were stained for CD4–APC-Cy7 (clone RM4-5), CD8-BV786 (clone 53-6.7), Thy1.2-BV605 (clone 30-H12) (all BD Biosciences), and p79/K^b^-PE (NIH Tetramer Core Facility, Emory University). Cells were sorted as CD4^–^CD8^+^Thy1.2^+^ p79/K^b+^ into 100% FBS (MidSci) using a FACSAria II Cell Sorter (BD Biosciences). Sorted samples were resuspended in RLT buffer (QIAGEN) and placed at –80°C. RNA was extracted using the RNeasy Micro Kit (QIAGEN) with on-column DNase digestion, and quality was assessed using a Fragment Analyzer 5300 (Agilent). A total of 1 ng of RNA was used as input for cDNA synthesis using the Clontech SMART-Seq v4 Ultra Low Input RNA Kit (Takara Bio) according to the manufacturer’s instructions. Amplified cDNA was fragmented and appended with dual-indexed barcodes using the Nextera XT DNA Library Prep Kit (Illumina). Libraries were validated by capillary electrophoresis on a TapeStation 4200 (Agilent), pooled at equimolar concentrations, and sequenced with paired-end 100 bp reads on an Illumina NovaSeq 6000, yielding approximately 25 million reads per sample. Alignment was performed using STAR version 2.9.7a (48), and transcripts were annotated using GRCm38_102. Transcript abundance estimates were calculated internal to the STAR aligner using the algorithm of htseq-count (49). Relative gene expression was visualized using raw counts normalized to CPM. Differential expression analysis was conducted using DESeq2 (v1.40.2) (50). GSEA of gene ontology pathways was conducted using the ClusterProfiler (v4.8.1) with Wald’s test followed by *P* value correction using the Benjamini-Hochberg procedure (51).

### Statistics.

All flow cytometry data were analyzed using FlowJo (v9, BD Biosciences). Absolute counts were calculated using CountBright Absolute Counting Beads (Invitrogen) according to the manufacturer’s instructions. Mann-Whitney *U* tests were performed for the analysis of 2 experimental groups. When 3 or more groups were present, data were analyzed by 1-way ANOVA followed by Tukey’s posttest. For intracellular cytokine staining, data were analyzed using 2-way ANOVA followed by Šidák’s correction for multiple comparison. Data are represented as the mean ± SEM. Results were considered significant if *P* < 0.05. All analyses were conducted using GraphPad Prism software (v9).

### Study approval.

This study was carried out in accordance with the recommendations in the *Guide for the Care and Use of Laboratory Animals* (National Academies Press, 2011). The protocol (PROTO201700558) was approved by the Emory University Institutional Animal Care and Use Committee.

### Data availability.

Individual values for summary data figures are available in the Supporting Data Values file. Next-generation sequencing data have been deposited in the NIH/National Center for Biotechnology Information Gene Expression Omnibus public database and assigned the accession number GSE293467.

## Author contributions

KLA designed, conducted, and analyzed experiments; interpreted data; and wrote and edited the manuscript. KBB conducted experiments and analyzed data. DL developed research models and conducted experiments. MLF conceived the study idea, designed experiments, interpreted data, and wrote and edited the manuscript.

## Supplementary Material

Supplemental data

Supporting data values

## Version 1 06/09/2025

Electronic publication
