# Supplementary material for: CD154:CD11b blockade enhances CD8+ T cell differentiation during infection but not transplantation
Source: JCI Insight. 2025 Jun 9;10(11):e184843. doi: 10.1172/jci.insight.184843 (PMC12220971; doi:10.1172/jci.insight.184843)
Supplement: Supplemental data [file jciinsight-10-184843-s008.pdf]

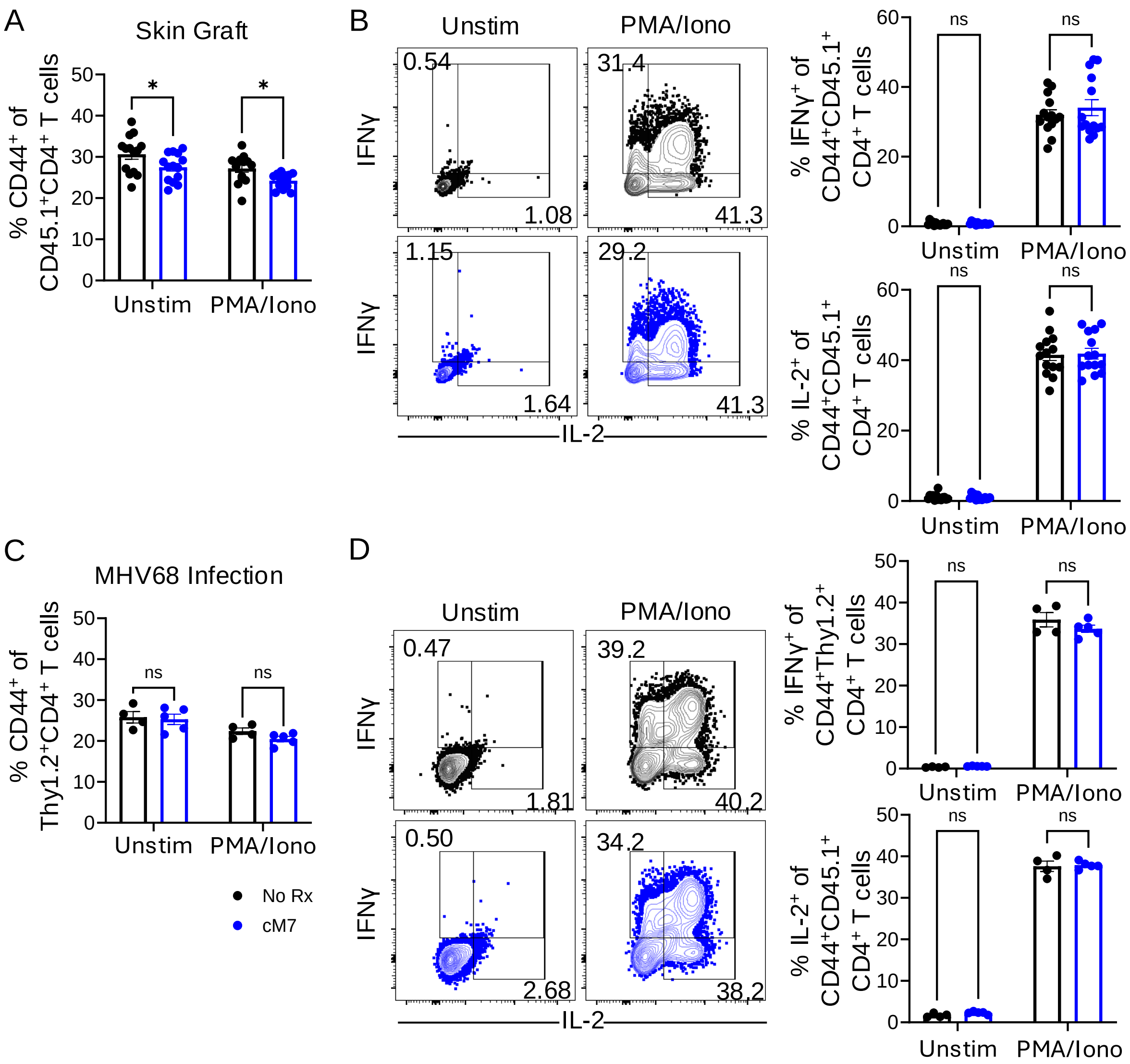

Supplement Figure 1. CD154:CD11b blockade did not impact CD4⁺ T cell effector function. CD45.1⁺ congenic mice were received Balb/c skin grafts and were untreated or treated with cM7 on days 0, 2, 4, and 6. Spleens were collected on day 10. A, Summary data of frequencies of CD44⁺ CD4⁺ T cells following restimulation with PMA and Ionomycin. B, Representative flow cytometry plots and summary data of frequencies of IFNγ- and IL-2-producing cells of CD44⁺ CD4⁺ T cells following restimulation with PMA and Ionomycin. Data are combined from 3 individual experiments with a total of 14 mice/group. WT C57BL/6 mice were infected with MHV68 and were untreated or treated with cM7 on days 0, 2, 4, and 6. Spleens were collected on day 14. C, Summary data of frequencies of CD44⁺ CD4⁺ T cells following restimulation with PMA and Ionomycin. D, Representative flow cytometry plots and summary data of frequencies of IFNγ- and IL-2-producing cells of CD44⁺ CD4⁺ T cells following restimulation with PMA and Ionomycin. Data are from one representative of 3 individual experiments with 4-5 mice/group. *$p < 0.05$, **$p < 0.01$ by two-way ANOVA with Sidak's test. ns, not significant

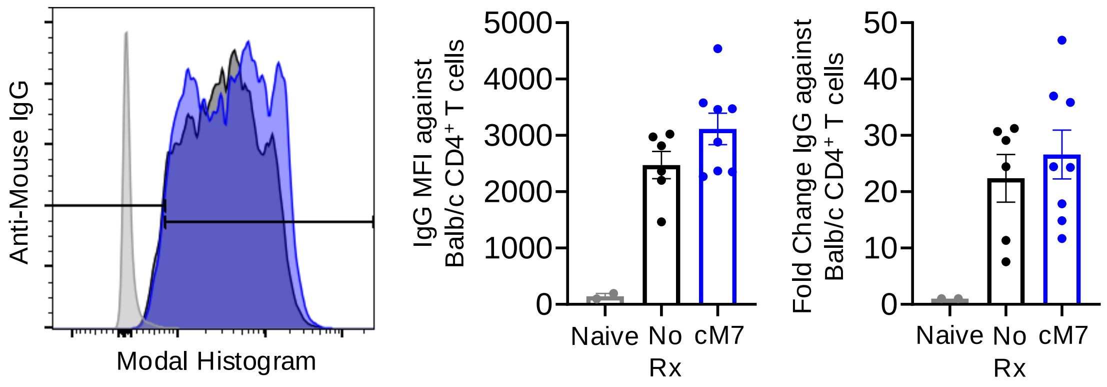

Supplement Figure 2. CD154:CD11b blockade does not significantly alter generation of donor-specific antibody. Serum was collected from wildtype B6 mice 14 days post-transplantation of Balb/c skin grafts. Flow-based crossmatch against Balb/c CD4$^+$ T cells was conducted and secondary stained for anti-mouse IgG. Representative histogram with mean fluorescence intensity (MFI) and fold change normalized to naïve control serum summary graphs are shown. Data are one representative replicate of 2 individual experiments with 5 mice/group each. Statistical analysis conducted by one-way ANOVA with Tukey's test.

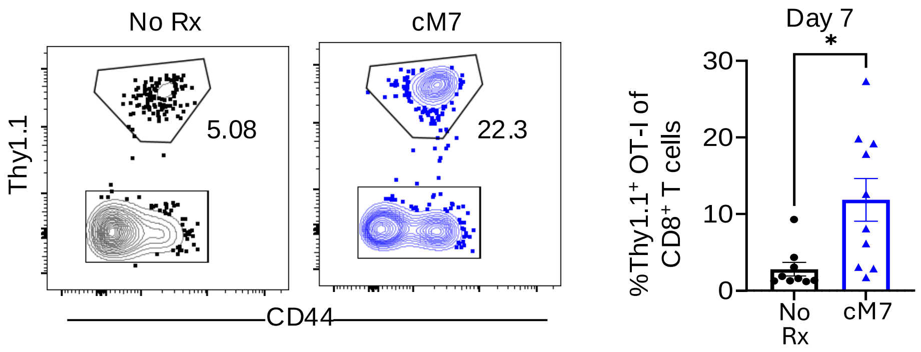

Supplement Figure 3. CD154:CD11b blockade-induced increase in antigen-specific CD8$^+$ cells is also observed in TCR transgenic OT-I T cells. WT C57BL/6 mice were infected with MHV68 and were untreated or treated with cM7 on days 0, 2, 4, and 6. Representative flow cytometry plots and summary data of frequencies of Thy1.1$^+$ OT-I T cells on Day 7 in the blood. Data are representative of 3 individual experiments with 5-10 mice/group. *$p < 0.05$ by Mann-Whitney test.

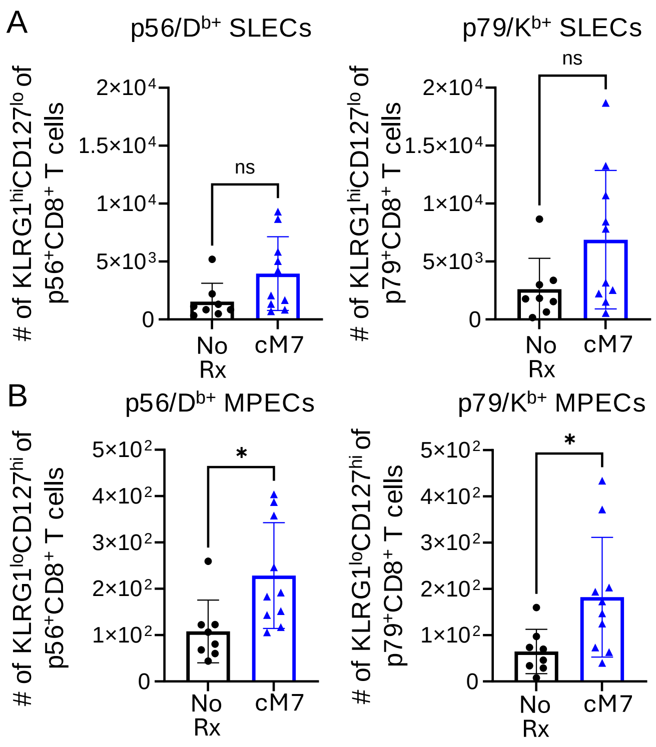

Supplement Figure 4. CD154:CD11b blockade resulted in an increase in absolute MPEC number among virus-specific CD8$^+$ T cell populations. WT C57BL/6 mice were infected with MHV68 and were untreated or treated with cM7 on days 0, 2, 4, and 6. A-B, Summary data of absolute number of KLRG1^{lo}CD127^{hi} MPECs and KLRG1^{hi}CD127^{lo} SLECs among antigen-specific CD8$^+$ T cells identified using MHC Class I tetramer for two lytic cycle viral epitope, p56 and p79, on day 10 in the blood are shown. Data are representative of 3 individual experiments with 8-10 mice/group. *$p < 0.05$ by Mann-Whitney test. ns, not significant

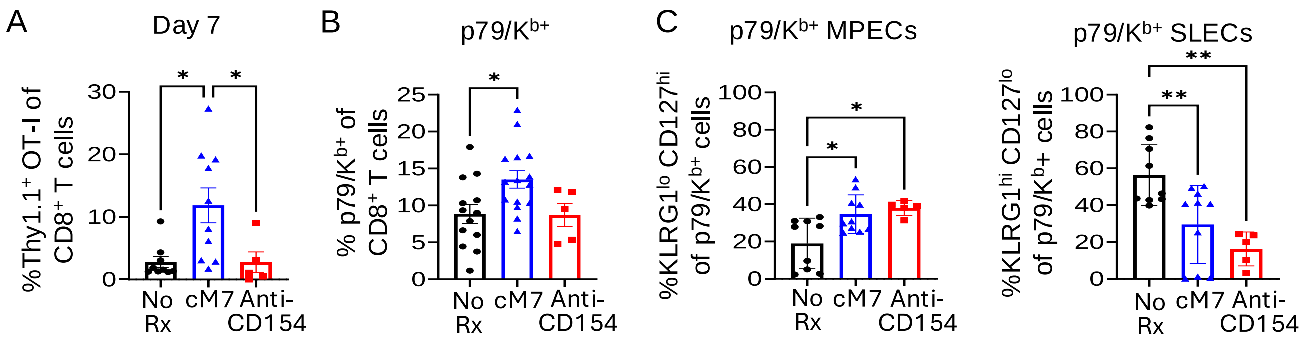

Supplement Figure 5. CD154:CD11b blockade-induced increase in antigen-specific $CD8^+$ cells is not observed, but altered differentiation is, with anti-CD154 treatment. WT C57BL/6 mice were infected with MHV68 and were untreated or treated with cM7 or anti-CD154 (clone MR1) on days 0, 2, 4, and 6. A, Summary data of frequencies of $Thy1.1^+$ OT-I T cells on Day 7 in the blood. B, Summary data of frequencies of antigen-specific $CD8^+$ T cells using MHC Class I tetramer for one lytic cycle viral epitope, p79, on Day 10 in the blood. C, Summary data of frequencies of $KLRG1^{lo}CD127^{hi}$ MPECs and $KLRG1^{hi}CD127^{lo}$ SLECs on day 14 in the spleen are shown. Data are representative of 2 individual experiments with 5-10 mice/group. *p < 0.05, **p < 0.01 by one-way ANOVA with Tukey's test.

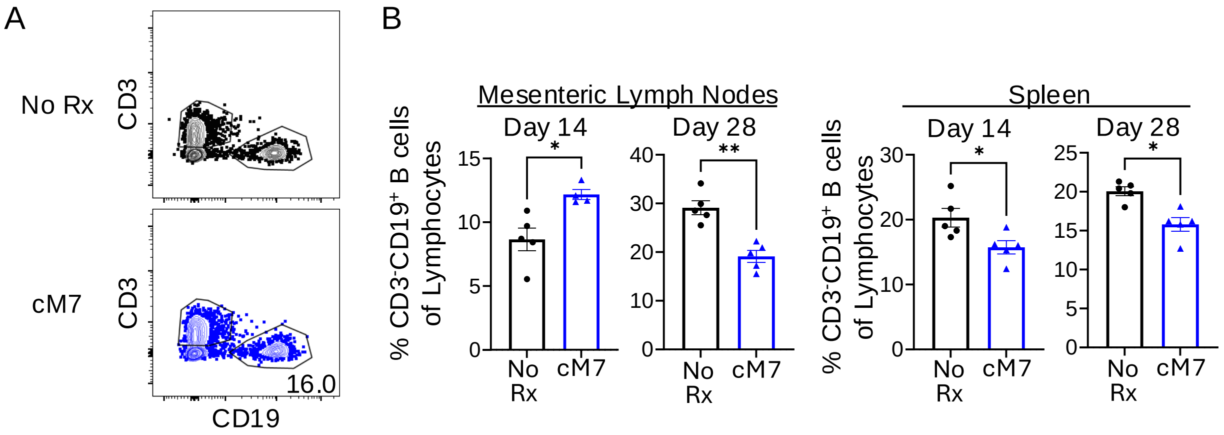

Supplement Figure 6. Bulk B cell replication is controlled during CD154:CD11b blockade. WT C57BL/6 mice were infected with MHV68-YFP and were untreated or treated with cM7 on days 0, 2, 4, and 6. Blood, mesenteric lymph nodes (mLN) and spleen were collected on days 14 or 28. Representative flow cytometry and summary data of $CD3^-CD19^+$ B cell frequencies of lymphocytes in the mLN and spleen on days 14 and 28. Data are one representative replicate of 2 individual experiments with 5 mice/group each. *$p < 0.05$, **$p < 0.01$ by Mann-Whitney Test.

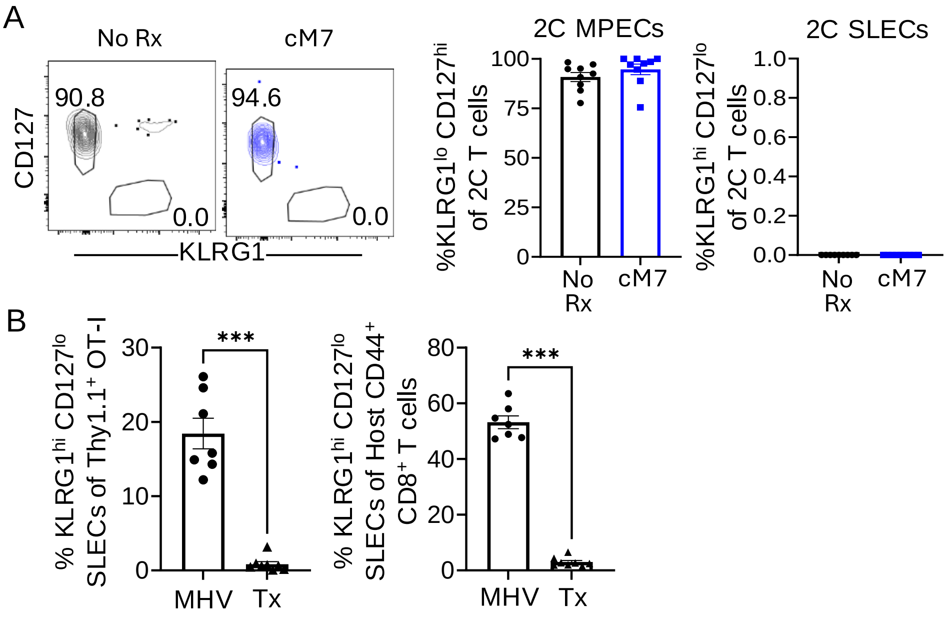

Supplement Figure 7. Disparate short-lived effector cell differentiation during infection versus transplantation are consistent in the direct comparison OT-I model. A, CD45.1+ congenic mice received 1×10^6 BALB/c-specific TCR transgenic 2C splenocytes and BALB/c skin grafts, and either no further treatment or cM7 on days 0, 2, 4, and 6. Spleens were collected 10 days post-transplantation. Representative flow cytometry plots and summary data of frequencies of KLRG1^{lo} CD127^{hi} MPECs and KLRG1^{hi} CD127^{lo} SLECs of 2C CD8^{+} T cells in the spleen. Data are representative of 3 individual experiments with 5-14 mice/group. B, WT C57BL/6 mice received TCR transgenic OT-I T cells and were infected with MHV68-OVA or received OVA-expressing skin grafts. Spleens were collected on day 10. Frequency of KLRG1^{hi} CD127^{lo} SLECs are shown for both OT-I and bulk host CD44^{+} CD8^{+} T cells. Data are one individual experiments with 7-8 mice/group. *$p < 0.05$, **$p < 0.01$, ***$p < 0.001$ by Mann-Whitney Test.
